# Time-Prolonged Release of Tumor-Targeted Protein–MMAE Nanoconjugates from Implantable Hybrid Materials

**DOI:** 10.3390/pharmaceutics14010192

**Published:** 2022-01-14

**Authors:** Naroa Serna, Aïda Falgàs, Annabel García-León, Ugutz Unzueta, Yáiza Núñez, Alejandro Sánchez-Chardi, Carlos Martínez-Torró, Ramón Mangues, Esther Vazquez, Isolda Casanova, Antonio Villaverde

**Affiliations:** 1 Institut de Biotecnologia i de Biomedicina, Universitat Autònoma de Barcelona, Cerdanyola del Vallès, 08193 Barcelona, Spain; srnaroa@gmail.com (N.S.); carlosmartineztorro@gmail.com (C.M.-T.); Esther.Vazquez@uab.cat (E.V.); 2 Departament de Genètica i de Microbiologia, Universitat Autònoma de Barcelona, Cerdanyola del Vallès, 08193 Barcelona, Spain; uunzueta@santpau.cat; 3 CIBER de Bioingeniería, Biomateriales y Nanomedicina (CIBER-BBN), 28029 Madrid, Spain; AFalgas@santpau.cat (A.F.); agarciale@santpau.cat (A.G.-L.); YNunez@santpau.cat (Y.N.); rmangues@santpau.cat (R.M.); 4 Biomedical Research Institute Sant Pau (IIB Sant Pau), Hospital de la Santa Creu i Sant Pau, 08025 Barcelona, Spain; 5 Josep Carreras Research Institute, Badalona, 08916 Barcelona, Spain; 6 Servei de Microscòpia, Universitat Autònoma de Barcelona, Bellaterra, 08193 Barcelona, Spain; Alejandro.Sanchez.Chardi@uab.cat; 7 Departament de Biologia Evolutiva, Ecologia i Ciències Ambientals, Facultat de Biologia, Universitat de Barcelona, Av. Diagonal 643, 08028 Barcelona, Spain

**Keywords:** protein nanoparticles, cytotoxic drugs, cancer treatment, drug depots, drug delivery, cancer cell targeting

## Abstract

The sustained release of small, tumor-targeted cytotoxic drugs is an unmet need in cancer therapies, which usually rely on punctual administration regimens of non-targeted drugs. Here, we have developed a novel concept of protein–drug nanoconjugates, which are packaged as slow-releasing chemically hybrid depots and sustain a prolonged secretion of the therapeutic agent. For this, we covalently attached hydrophobic molecules (including the antitumoral drug Monomethyl Auristatin E) to a protein targeting a tumoral cell surface marker abundant in several human neoplasias, namely the cytokine receptor CXCR4. By this, a controlled aggregation of the complex is achieved, resulting in mechanically stable protein–drug microparticles. These materials, which are mimetics of bacterial inclusion bodies and of mammalian secretory granules, allow the slow leakage of fully functional conjugates at the nanoscale, both in vitro and in vivo. Upon subcutaneous administration in a mouse model of human CXCR4^+^ lymphoma, the protein–drug depots release nanoconjugates for at least 10 days, which accumulate in the tumor with a potent antitumoral effect. The modification of scaffold cell-targeted proteins by hydrophobic drug conjugation is then shown as a novel transversal platform for the design of slow releasing protein–drug depots, with potential application in a broad spectrum of clinical settings.

## 1. Introduction

**Figure 1:**
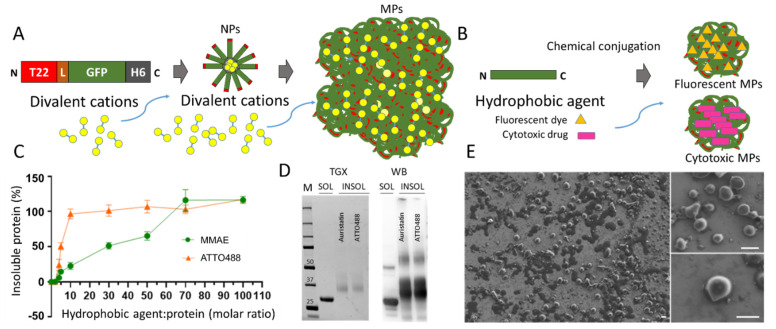
Fabrication of protein–drug microscale depots. (**A**). The modular protein T22-GFP-H6 has been used in this study as a model. T22 (red box) is a cationic peptide targeting CXCR4 [53], L (brown box) is a peptidic linker (GGRSSRSS) that provides flexibility, GFP (green box) is an enhanced GFP and H6 is a hexahistidine tag (black box) [54,55]. H6 is useful for both chromatographic purification of the protein [54,55,56,57] and cation-mediated assembly [55,58]. N and C indicate the amino and carboxy-terminal ends, respectively. His-tagged proteins such as T22-GFP-H6 can be organized as progressively complex supramolecular structures (NPs and MPs) through exposure to increasing amounts of divalent cations [59] (yellow circles). Upon addition of divalent cations at molar excess, NPs aggregate as MPs of around 2–3 microns, called artificial IBs [29]. (**B**). In the concept presented here, a generic polypeptide (in green), can be aggregated as MPs by means of chemically attaching hydrophobic molecules. Thus, the formation of MPs would be achieved irrespective of histidine-rich tails and divalent cations, by using either fluorescent dyes such as ATTO488 (yellow symbol) or cytotoxic drugs (pink symbol) such as MMAE, provided they are hydrophobic. (**C**). Progressive, dose-dependent precipitation of nanoconjugates under increasing doses of the hydrophobic chemical. (**D**). Detection of soluble T22-GFP-H6 and insoluble protein–agent conjugates by TGX band staining and by anti-H6 immunodetection upon SDS-PAGE. (**E**). FESEM determination of T22-GFP-H6- MMAE MPs. Bars represent 500 nm.

Reaching time-sustained drug delivery upon a single-dose administration is of paramount clinical relevance in pathologies, such as cancer, that require steady levels of the drug at the target organs over significant periods of time [1,2,3,4]. Repetitive intravenous drug administration results in large fluctuations of drug concentration (which could reach toxic levels at the peaks and be subtherapeutic at the valleys). Hence, a diversity of materials organized as drug-holding matrices are under development, aiming to achieve the progressive time-sustained release of the payload drug [5]. Such dynamic depots are usually based on biocompatible, polymeric materials with fibrous architectures that hold the embedded drug in inner spaces, aiming for either passive or stimuli-based release [5,6,7,8,9]. However, reaching steady drug levels is particularly difficult for the small-molecular-weight cytotoxic agents used in the chemotherapy of cancer. This is due to their poor retention in such scaffolding depots and because of the renal clearance of the drug below approximately 7 nm [10,11,12,13]. Among emerging drug-releasing materials, bacterial inclusion bodies (IBs) are natural protein depots, at the micro scale, that show intriguing secretion properties of the IB-forming protein [14]. In the cytoplasm of recombinant bacteria, IBs are observed as clusters of folded, partially folded and unfolded protein versions that contain undefined traces of entrapped bacterial molecules [15]. In bacterial cells, IBs are targets of the bacterial quality control for continuous protein extraction to carry out refolding or proteolysis [16,17,18]. However, when extracted from producing bacteria, IBs have been successfully explored as the local [19] and remote secretors [20] of protein-only drugs in cancer. However, since the accompanying bacterial molecules (namely, proteins, nucleic acids and cell wall components) might be harmful in clinical settings, chemically pure protein microscale granules have been developed as IB mimetics [21]. This is achieved by the in vitro controlled clustering of pure protein through the coordination of divalent cations [22] with histidine-rich tails (usually the poly-histidine H6) overhanging from the protein (Figure 1A). Under physiological conditions, both natural IBs and their artificial versions mimic the secretory granules of the endocrine system that release peptidic hormones from Zn-supported amyloidal clusters [23,24,25,26]. Both IB versions are then self-disintegrating protein-only materials [27] suited for time-sustained release of protein drugs [28,29]. Because of the nature of such protein aggregates, the application of this concept and technology has been so far restricted to protein-only drugs.

In this study, we have approached, for the first time, the fabrication of artificial IBs with the assistance of chemical drugs covalently coupled to a pure protein. This has been achieved by mimicking the in vivo IB-formation process, which is partially mediated by hydrophobic protein patches, through exploiting drug hydrophobicity as an in vitro aggregation-prone agent. The resulting protein-based materials, aggregated upon coupling hydrophobic drugs, act, similarly to IBs, as efficient secretory granules for the systemic slow release of protein–drug nanoconjugates. This concept has been demonstrated by using a self-assembling, tumor-targeted protein as a model and a small antitumoral cytotoxic drug, namely Monomethyl Auristatin E (MMAE), as the hydrophobic and aggregation agent. Based on this principle, the concept of IB-like secretory granules can be expanded through the convenient chemical modification of the forming protein as an early step in the fabrication process.

## 2. Materials and Methods

### 2.1. Protein Production and Purification

The synthetic gene encoding the protein T22-GFP-H6 had been provided by Geneart (Thermo Fisher Scientific, Waltham, MA, USA), as inserted in the expression vector pET22b (Novagen, Merck Millipore, Burlington, MA, USA). T22-GFP-H6 is a modular protein that contains the peptide T22, an enhanced GFP protein and a hexahistidine tail (H6) (Figure 1A). This protein tends to spontaneously self-assemble as NPs upon purification, by recruiting traces of divalent cations from the media [30]. T22 is a short cationic peptide that acts a tumor-homing agent, as it binds the cell-surface cytokine receptor CXCR4 and efficiently internalizes CXCR4^+^ cells [31]. Since CXCR4 is overexpressed in more than 20 human cancers, this marker is an excellent target for selective cancer therapies [32]. The fusion protein was produced in *Escherichia coli* Origami B strain (Novagen) overnight at 20 °C upon the addition of 0.1 mM isopropyl-β-D-thiogalactopyronaside (IPTG) to induce the recombinant gene expression from the expression vector. Bacterial cells were harvested by centrifugation (10 min at 5000× *g*) and resuspended in a Tris buffer (20 mM Tris, 500 mM NaCl, 10 mM Imidazole, pH = 8) in the presence of EDTA-Free protease inhibitor (cOmplete EDTA Free, Roche, Basel, Switzerland). Cells were then disrupted in an EmulsiFlex C5 cell disruptor (Avestin, Biopharma, UK) by 2 sequential rounds at 8000–10,000 *psi* and the soluble fraction was separated by centrifugation (45 min at 20,000× *g*). The soluble protein fraction was then purified by immobilized metal affinity chromatography (IMAC) using a nickel-loaded HisTrap HP 1 mL column in an ÄKTA Pure FPLC (Cytiva, Marlborough, MA, USA). Protein elution was achieved by a linear gradient of elution buffer (20 mM Tris, 500 mM NaCl, 500 mM Imidazole, pH = 8). The obtained protein was then dialyzed against a sodium carbonate with salt buffer (166 mM NaCO_3_H, 333 mM NaCl, pH = 8). Protein purity was finally evaluated by SDS-PAGE and Western-blot immunodetection using an anti-His mouse monoclonal antibody (Santa Cruz Biotechnology, Santa Cruz, CA, USA), while its integrity was determined by MALDI-TOF mass spectrometry. The amount of obtained protein was determined by the conventional Bradford assay.

### 2.2. Drug Conjugation and Generation of Microparticles

T22-GFP-H6 was covalently conjugated to the fluorescent marker ATTO488 (commonly used for fluorescent protein labelling) or the antineoplastic drug MMAE (both hydrophobic) through protein lysine-amines by amide or alquilamine bound generation, respectively. For this, NHS-Ester-functionalized ATTO488 (Sigma-Aldrich, Madrid, Spain) or maleimidocaproyl-functionalized MMAE (Levena Biopharma) molecules were incubated in the presence of T22-GFP-H6 in a range of different molar ratios (0–100 fold molar excess) for 1 h at room temperature. Under these conditions, T22-GFP-H6 occurs as spontaneously formed protein-only nanoparticles (NPs) of around 12 nm, that keep the CXCR4-targeting properties of T22 [33]. Then, protein precipitation and the consequent generation of larger-order microparticles (MPs) was monitored by centrifugation (15 min at 15,000× *g*) and the remaining soluble protein was quantified by Bradford assay. The protein integrity and conjugation efficiency was finally determined in the formed MPs by protein band shift analysis in an SDS-PAGE and subsequent protein immunostaining by Western blot, using a mouse monoclonal anti-His antibody (GenScript).

### 2.3. Release of Soluble Nanoconjugates

The release of soluble protein from T22-GFP-H6-MMAE MPs was analyzed upon incubation in sodium carbonate with salt buffer at 37 °C for different times (up to 8 days, d). For this, the remaining T22-GFP-H6-MMAE MPs were separated from soluble protein by centrifugation (15 min at 15,000× *g*) and the soluble fraction was quantified by Bradford assay. The purity and integrity of the released protein material was then analyzed by SDS-PAGE and Western blot using a mouse anti-His monoclonal antibody (GenScript). The MMAE payload in the released nanoconjugates was finally analyzed by MALDI-TOF mass spectrometry.

### 2.4. Morphometric Characterization

The nanoscale morphometry (size and shape) of T22-GFP-H6-MMAE MPs and of the released T22-GFP-H6-MMAE nanoconjugates was visualized at a nearly native state with a Field Emission Scanning Electron Microscope (FESEM). Samples in buffer were deposited 1 min in silicon wafers (Ted Pella, Inc Redding, CA, USA), air dried and immediately observed in a FESEM Merlin (Zeiss, Oberkochen, Germany) operating at 1 KV and equipped with both standard and *in-lens* secondary electron detectors. Representative images of MPs and nanoconjugates were collected at three magnifications (MPs: 10,000×, 70,000×, 100,000×; Nanoconjugates: 70,000×, 240,000×, 370,000×). The size distribution of the released protein was determined by dynamic light scattering (DLS) in a Zetasizer Nano ZS (Malvern Instruments Ltd., Malvern, UK) at 633 nm. Average values were obtained after the independent measurement of protein samples in triplicate.

### 2.5. Cell Culture, Protein Internalization and Competition Assays

HeLa cells (ATCC, CCL-2) were incubated in 24-well plates in MEM alpha medium (Gibco, Rockville, MD, USA) containing 10% fetal bovine serum (Gibco) in a humidified atmosphere and 5% CO_2_ at 37 °C. At the appropriate density, plates were washed with DPBS (Gibco), culture medium substituted by a serum-free Optipro medium (Thermo Fisher Scientific) supplemented with 2 mM L-glutamine and incubated in the presence of 100 nM of T22-GFP-H6-ATTO488 MPs for 2 h. A potent CXCR4 receptor antagonist AMD3100 (octahydrochloride hydrate, Sigma-Aldrich) or non-conjugated T22-HSNBT-H6 NPs were incubated 1 h before sample addition for competition assays. The plates were then washed with DPBS (Gibco), treated with a “harsh” trypsin digestion (1 mg min^−1^ for 15 min) to remove externally attached proteins. Fluorescence or internalized materials were finally analyzed in a FACS Canto flow cytometer (Becton Dickinson, Franklin Lakes, NJ, USA) using a 488 nm laser and a 530/30 bandpass filter detector. All samples were analyzed in duplicate, and the results are expressed as mean fluorescence ± standard error.

### 2.6. In Vitro Cell Viability Assay

CXCR4^+^ HeLa cells (ATCC, CCL-2), CXCR4^+^ SW1417 cells (ATCC-CCL-238) and CXCR4^+^ PANC-1 cells (ATCC-CCL-1469) were incubated in opaque 96-well plates in 90 µL of MEM alpha medium (Gibco) containing 10% fetal bovine serum (Gibco) in a humidified atmosphere and 5% CO_2_ (HeLa and PANC-1) or 10% CO_2_ (SW1417) at 37 °C. Then, from 25 nM to 2 µM of T22-GFP-H6-MMAE MPs or 1 µM of T22-GFP-H6-MMAE nanoconjugates were added and incubated for 48 h (HeLa and PANC-1) or 72 h (SW1417). Cell viability was finally tested by CellTiter-Glo^®^ Luminescent Cell Viability Assay (Promega) in a Victor 3 luminescent plate reader (PerkinElmer). All samples were analyzed in triplicate and data expressed as mean % of viability (related to control cells) ± standard error.

### 2.7. Animal Maintenance

All experimental procedures were reviewed and approved by the Hospital de la Santa Creu i Sant Pau Animal Ethics Committee. The animal experiments were carried out according to European Council directives for welfare of the laboratory animals. Four-week-old female Swiss nude mice were obtained from Charles River Laboratories (L-Abreslle, France) and maintained in specific pathogen-free (SPF) conditions with sterile food and water ad libitum. All in vivo procedures were conducted in accordance with the guidelines approved by the institutional animal Ethics Committee of Hospital Sant Pau (project number 10108 by the Government of Catalonia) and performed following the European Union Directive 2010–63-EU for the welfare of the laboratory animals.

### 2.8. In Vivo Biodistribution and Antineoplastic Effect of T22-GFP-H6-MMAE MPs

To generate the diffuse large B-cell lymphoma (DLBCL) subcutaneous (SC) mouse model, 10^7^ U2932 cells were injected subcutaneously into the dorsal left flank of Swiss Nude mice. Tumor growth was monitored three times per week with a Caliper (tumor volume = width^2^ × length/2). When tumors reached 120–200 mm^3^, mice received a single SC dose in the distal region of the tumor of 1 mg T22-GFP-H6-MMAE MPs dissolved in 100 μL of sodium carbonate buffer with salt or solely 100 μL of buffer in control mice (n = 1/group). Fluorescence intensity (FLI) was measured ex vivo at different time points (0 h, 5 h, 24 h, 48 h, 5 and 10 days) using the IVIS Spectrum 200 Imaging System (PerkinElmer, Waltham, MA, USA). Animal weight was registered along the experimental time. FLI, expressed as average radiant efficiency, was detected by the fluorescence emission of the GFP domain of T22-GFP-H6-MMAE MPs, for measuring accumulation of MPs. FLI from experimental mice was calculated subtracting the FLI autofluorescence of mice injected only with buffer. Finally, the injection point, the tumors and all organs were collected, fixed in 4% formaldehyde and included in paraffin for histological and immunohistochemical assays.

### 2.9. Histopathology Analysis

Hematoxylin and eosin (H&E) staining was performed in paraffin-embedded organs to evaluate the possible off-target toxicity of the MPs (kidney, liver, spleen, and bone marrow). Images of the histological sections were taken at 400× using an Olympus DP73 digital camera. They were further processed with the cellSens Dimension 1.9 software (Olympus) and analyzed by two independent observers.

### 2.10. Assessment of Apoptotic Cell Death

The mechanism of cell death caused by the treatment with T22-GFP-H6-MMAE MPs was evaluated by staining with the DNA-specific fluorescent probe 4′,6-diamidino-2-phenylindole (DAPI). Tumor tissue sections were dried at 60 °C for 1 h, rehydrated through decreasing concentrations of alcohol, permeabilized with Triton X-100 (0.5%) and mounted with DAPI dye (ProLong™ Gold Antifade Mountant with DAPI, P36935, Invitrogen, Carlsbad, CA, USA). Then, stained slides were visualized using a fluorescence microscope (Olympus BX53, Olympus, Tokyo, Japan) and representative pictures were taken using an Olympus DP73 digital camera (400×). Mitotic catastrophe or apoptotic bodies were quantified as cell death events using the cell counter tool in Image J software (1.8.0.172) in 5 random fields of each condition.

### 2.11. Immunohistochemistry (IHC)

Anti-H2AX pSer139 (γH2AX, 1:800, Novus, Centennial, CO, USA) antibody was used in mouse paraffin-embedded tumors to determine DNA damage in the treated samples. IHC analyses were performed in a DAKO Autostainer Link48 following the manufacturer’s instructions. Representative images were obtained using an Olympus DP73 digital camera, and the γH2AX determination was carried out with the Image J software (1.8.0.172) with the “Colour Deconvolution Plugin” by adjusting the threshold to 100 and the “Analyze Particles Plugin” for detecting all stained areas. The total area was obtained using the ROI Manager.

### 2.12. Statistical Analysis

All numerical results are expressed as mean ± standard error (SE). Differences between groups were analyzed using unpaired *t*-Student test through the GraphPad Prism 8.0.2 software. Differences were considered statistically significant at *p* ≤ 0.05.

## 3. Results and Discussion

The technologies of protein clustering based on divalent cations rely on histidine-rich peptides overhanging from engineered proteins [22]. Then, they cannot be applied to generate depots of chemicals, which for small-molecular-weight antitumoral drugs would be of obvious interest. Interestingly, many antitumoral drugs are hydrophobic, and on the other hand, solvent-exposed hydrophobic protein patches are well known drivers of protein aggregation [34,35,36,37]. In this regard, the genetic fusion of hydrophobic peptides to essentially soluble proteins promotes the immediate and efficient in vivo aggregation of the whole fusion [38]. Therefore, we wondered if covalently attaching hydrophobic drugs to multifunctional proteins in vitro would also promote the controlled aggregation of protein–drug conjugates in a controlled way (Figure 1B). Additionally, we speculated that the resulting materials might release such conjugates under physiological conditions, as occurs with bacterial IBs [28,39] or other types of IB-like protein aggregates [29]. Following this idea, we have explored this hypothesis by cross-linking the anticancer drug MMAE (a highly hydrophobic small molecular weight molecule) with the tumor-targeted functional protein T22-GFP-H6.

T22-GFP-H6 is a GFP-containing modular protein (Figure 1A) that, assisted by divalent cations in the media [30], assembles as fluorescent NPs [33]. Previous analyses by DLS and other analytical methods indicated that T22-GFP-H6 NPs are homo-oligomers of around 12 nm in size [33]. Because of the CXCR4-binding peptide T22 [40,41], which is solvent exposed in the oligomers [33], the assembled protein targets and penetrates CXCR4-overexpressing cancer stem cells upon systemic administration in cancer models [42]. Therefore, these protein materials have been proven to be efficient drug carriers in different types of cancers [43,44,45,46,47]. The chemically induced aggregation of T22-GFP-H6 was firstly explored by conjugating increasing amounts of the hydrophobic dye ATTO488 (Figure 1B). This molecule is widely used as reporter in biodistribution studies [48] and easy to monitor by its fluorescent emission in a range distinguishable from that of GFP [49].

As observed, ATTO488 was a dose-dependent inducer of protein aggregation, causing an almost full precipitation of the protein in the sample when coupling the dye at a drug:protein ratio of 10 (Figure 1C). This result encouraged us to test the hydrophobic drug MMAE, used as a front line in the treatment of several cancers. Being highly cytotoxic, this drug is only developed in form of antibody–drug conjugates to ensure selective cell destruction, as the drug alone would cause severe systemic toxicity [45,50,51,52]. As in the case of ATTO488, MMAE promoted the aggregation of T22-GFP-H6 in a more progressive way than the dye, causing the almost full protein aggregation at a molar drug:protein ratio of 70 (Figure 1C). In both cases, molar ratios over 1 were clearly necessary for protein aggregation, and the delayed effect of the drug prompted us to envisage a highly controllable conjugation process. Interestingly, the two categories of protein aggregates contained full-length T22-GFP-H6 with retarded motility in the electrophoresis gel (Figure 1D). The band shift of the entire protein indicated that the aggregation process did not compromise its proteolytic or structural stability and proved an efficient crosslinking between the anticancer drug and the protein.

At this point, we wondered if the chemical conjugation at high doses of the chemicals resulted in a mere protein–drug amorphous deposit or instead, in a structurally organized material. Both natural IBs [39] (in whose formation, hydrophobic protein patches are involved [60]) and their mimetic secretory granules (constructed by cation-mediated protein crosslinking [22]) range from 1 to 3 microns in diameter. Additionally, these particles show both pseudo-spherical morphologies. Similarly, the T22-GFP-H6-ATTO488 clusters formed at a molar ratio 1:100 organized as pseudospherical and highly regular entities that exhibited an IB-like smooth surface (Figure 1E), being indicative of an ordered deposition process.

**Figure 2:**
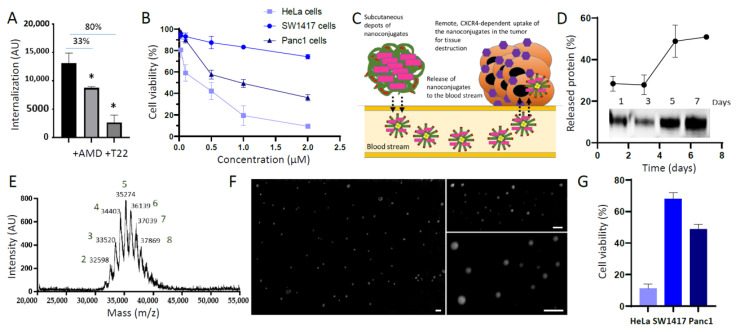
Release of CXCR4-targeted cytotoxic nanoconjugates from protein–drug depots. (**A**). Internalization of T22-GFP-H6-ATTO488 MPs into HeLa cells in absence or in presence of the CXCR4 antagonist AMD3100 (AMD) or soluble T22-GFP-H6 (T22). AU are arbitrary fluorescence units. (**B**). Dose-dependent viability of three different CXCR4^+^ cell lines upon exposed to T22-GFP-H6-MMAE MPs for 48 h (HeLa and Panc-1) and for 72 h (SW1417). (**C**). A model for the expected release of nanoconjugates from MP depots and remote targeting to and destruction of CXCR4-overexpressing cancer cells. MPs (around 2–3 microns) formed by T22-GFP-H6-MMAE (green and purple) would release T22-GFP-H6-MMAE NPs (around 11 nm) to the blood stream, in a way similar to that observed in T22-GFP-H6 IBs [65]. Since T22-GFP-H6 tends to assemble with the assistance of divalent cations from the media, T22-GFP-H6-MMAE are expected to occur as NPs. Because of the peptide T22 (red symbols), a ligand of CXCR4 (purple hexagonal symbols), these materials might accumulate intracellularly in CXCR4-overexpressing cells, as observed for unconjugated T22-GFP-H6 NPs [31]. This fact would allow a remote but selective destruction of the CXCR4^+^ tumor tissue. Symbols are as in Figure 1A,B). (**D**). Determination of protein conjugates released in vitro in a physiological buffer from T22-GFP-H6-MMAE MPs, through Western blot (inset) and densitometry. (**E**). MALDI-TOF analysis of released conjugates collected after 24 h of incubation in vitro. Numbers above the molecular masses indicate the corresponding number of MMAE molecules per polypeptide. (**F**). Ultrastructural characterization of the released nanoconjugates (T22-GFP-H6-MMAE NPs) collected after 24 h of MPs incubation in vitro. Size bars represent 100 nm. (**G**). Viability of three different CXCR4^+^ cell lines upon exposure to T22-GFP-H6-MMAE NPs for 48 h (HeLa and Panc-1) and for 72 h (SW1417). The tested material was collected after 24 h of MP incubation in vitro. * *p* ≤ 0.05.

These promising observations prompted us to further explore the functionalities of the protein–drug conjugates in such a packaged form and to specifically evaluate if both the CXCR4 targeting by T22 and the cytotoxicity of MMAE could have been combined for a selective cell killing effect. Like T22-containing IBs [61], T22-empowered protein-MMAE granules internalized HeLa cells in a CXCR4-dependent way (Figure 2A), and as such uptake was inhibited by the CXCR4 antagonist AMD3100 [62,63,64] and by soluble uncoupled T22-GFP-H6 (Figure 2A). Additionally, the exposure to MMAE-containing granules induced cell death in several CXCR4^+^ cancer cell lines (Figure 2B), which is indicative of the availability of functional forms of the drug upon internalization.

At this stage, we questioned if the MPs produced by drug binding could release, in a time-prolonged way, CXCR4-targeted protein–drug conjugates. Additionally, more specifically, if due to the cation-assisted self-assembling properties of T22-GFP-H6 [30], such conjugates would be nanostructured. If so, the microscale granules could be used as a source of nanoconjugates for remote and selective tumor tissue destruction, as pictured in Figure 2C. Administered subcutaneously, T22-GFP-H6-MMAE MPs would be expected to release nanoscale protein–drug conjugates to the bloodstream. Because of the CXCR4 targeting conferred by its highly selective ligand T22, they should accumulate, intracellularly, in CXCR4-overexpressing tumor tissues for their destruction. In fact, such a remote delivery has previously been observed through bacterial IBs [65] and cation-derived artificial IBs formed by tumor-targeted toxins [29]. To validate this hypothesis, we first determined that T22-GFP-H6-MMAE MPs, placed in physiological buffer, progressively released conjugated protein at least during 7 days, at that time where only about 40% of the initial protein remained (Figure 2D). The MALDI-TOF analysis of the released protein revealed the occurrence of one to eight MMAE molecules attached to each protein molecule, peaking at five drug molecules per polypeptide (Figure 2E). Additionally, and very importantly, when analyzing the size of the released protein–drug nanoconjugates by DLS (not shown), they were found assembled in form of NPs of around 11 nm, fitting to microscopy observations (Figure 2F). This size was, in fact, very similar to that observed when exploring plain unconjugated T22-GFP-H6 [33]. These leaked nanoconjugates were tested for cell killing over the three CXCR4^+^ cell lines used in above experiments, showing a differential cytotoxicity (Figure 2G). Interestingly, the cytotoxicity profile of the NPs was precisely matching that of the whole MPs (compare to Figure 2B), proving that the pharmacological action of the CXCR4-targeted MMAE remained unchanged irrespective of the micro- or nano-formulation of the protein–drug conjugates.

**Figure 3:**
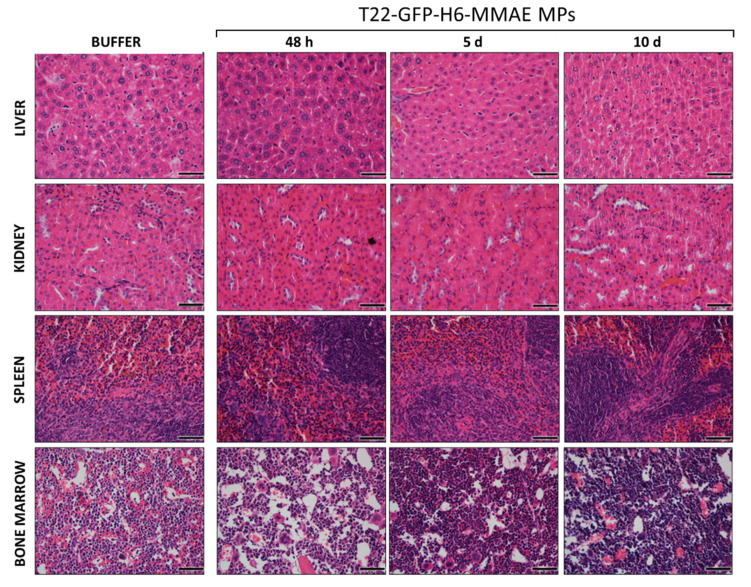
Toxicity analysis of T22-GFP-H6-MMAE MPs in a SC CXCR4^+^ DLBCL mouse model. H&E staining of the liver, kidney, spleen and bone marrow of mice treated with buffer or MPs at 48 h, 5 and 10 days after the treatment. Pictures were taken at 400× (scale bars = 50 μm).

**Figure 4:**
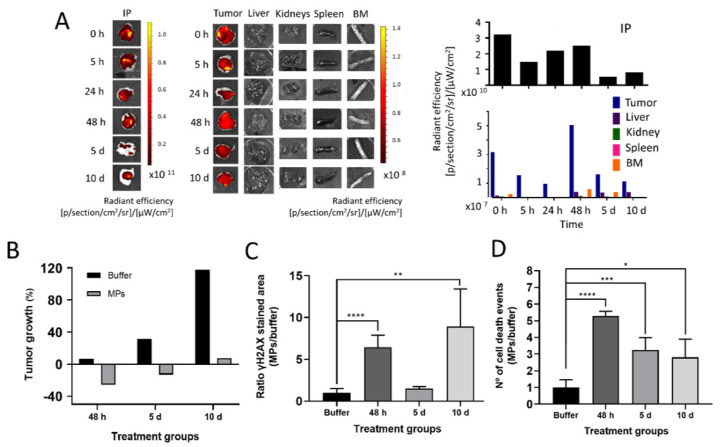
In vivo biodistribution and antitumoral effect of subcutaneously administered protein-MMAE depots in a SC mouse model of CXCR4^+^ DLBCL^−^ cells. (**A**). Ex vivo fluorescence imaging (left) and numerical representation of FLI (right) at the injection point (IP) and tumor, liver, kidneys, spleen and bone marrow (BM) upon remote subcutaneous administration of 1 mg of T22-GFP-H6-MMAE MPs in SC CXCR4^+^ DLBCL mouse model. Different recording times are shown from 5 h to 10 days. Fluorescence intensity (FLI) was calculated subtracting the autofluorescence of buffer-treated mice and represented as radiant efficiency. (**B**). Tumor volume variation relative to time 0 at 48 h, 5- and 10 days post administration of T22-GFP-H6-MMAE MPs. (**C**). Quantification of the IHC-positive stained tumor tissue area marked by γH2AX at 48 h, 5 and 10 days after the administration of T22-GFP-H6-MMAE MPs. The ratio quantification was obtained by dividing the area of positive cells in 5 counted fields in MP-treated samples by the mean of the positive area in 5 fields of buffer-treated samples. (**D**). Quantification of cell death events by DAPI staining in tumor tissues at different times post administration of T22-GFP-H6-MMAE MPs. The ratio quantification was obtained by dividing the number of cells undergoing MC or apoptosis in 5 fields of MPs-treated samples (48 h, 5 and 10 days) by 5 fields of buffer-treated samples. * *p* ≤ 0.05; ** *p* ≤ 0.01; *** *p* ≤ 0.001; **** *p* ≤ 0.0001.

At this point, we wondered if the generated MPs could be used in vivo as depots for the time-prolonged release of CXCR4-targeted nanoconjugates. For this, we administered 1 mg of T22-GFP-H6-MMAE MPs, subcutaneously, in a contralateral place remote to tumor, in an SC mouse model of CXCR4^+^ human lymphoma. The fluorescence at the injection point and at the tumor, liver, kidneys, spleen and bone marrow (BM) was monitored during the experimental time (up to 10 days), in the absence of systemic toxicity (Figure 3). As observed (Figure 4A), the fluorescence emission at the injection point decreased over time, concomitant to the appearance of fluorescence in tumor. The tumor signal peaked around 48 h post administration but was still detectable after 10 days (Figure 4A). By contrast, only negligible fluorescence levels were observed in liver, kidneys, spleen and BM from 5 h to 10 days after T22-GFP-H6-MMAE MP administration (Figure 4A). Such a tumor accumulation and the low protein uptake by off-target organs, together with the absence of local or systemic toxicity (Figure 3), were indicative of the chemical drug being stably attached to the protein and of the nanoconjugate being slowly released from the depot in a fully tumor-targeted way. It was also indicative of the absence of renal filtration that would have damaged renal tissues. Additionally, the fluorescent signal indicated that the protein remained stable and properly folded both in the depot version but also when released as soluble conjugated materials.

In addition, the dramatic arrest of the tumor growth observed in treated animals (Figure 4B) confirmed the activity of MMAE in the CXCR4-targeted nanoconjugates. The analysis of γH2AX (a marker of DNA damage) (Figure 4C) and specially the number of dead cell bodies in tumor (Figure 4D) (that peaked at 48 h, coincident with the maximum accumulation of the material, Figure 4A) fully supported the antitumoral potential of the drug–protein complexes once released from the SC depot.

The present study represents an early proof of concept that would of course benefit from further support through a broader catalogue of hydrophobic chemicals. However, the results presented here using ATTO488 and MMAE are extremely robust. These data point to a novel method for the fabrication and administration of depots for the delivery of tumor-targeted nanoconjugates. The possibility to combine small-molecular-weight cytotoxic chemicals with tumor-targeted nanoparticles offers an unusual possibility to slowly release safe and potent protein–drug complexes during prolonged time periods, in a new biomedical concept. Inspired by an emerging methodology based on His-Zn coordination [29,58], the proposed principle uses the hydrophobic character of some chemicals as a potent aggregation tool (Figure 1C). This allows us to develop a new type of depot that is especially appropriate for the sustained administration of highly cytotoxic drugs (Figure 1A), which can be only tolerated under strict targeting. The high drug-loading capacity (Figure 2E) of the depots together with the stability (Figure 2D) and effectiveness (Figure 4C,D) of the released complexes make the proposed system a potent and unusual platform to be explored for prolonged cancer treatments.

## 4. Conclusions

We have demonstrated the feasibility of protein-based, chemically hybrid microscale depots for the slow release of a drug attached to a scaffold protein. This was achieved by exploiting the hydrophobic character of the protein-linked drug in a simple fabrication protocol, whose products mimic some main properties of bacterial IBs. After a single-dose depot administration, a drug-loaded tumor-targeted protein reach the target tissue in form of nanoconjugates, upon leaking from such depots. The tissue destruction achieved by such nanoconjugates is highly selective, proving a very robust linkage of the protein and drug in any of the forms assumed by the complex (namely MPs and NPs). This is so even for a drug that, like MMAE, is extremely cytotoxic. The complete set of in vitro and in vivo results presented here, using ATTO488 and the antitumoral drug MMAE, demonstrates that such a concept can be successfully applied to the design of novel cancer treatments for the sustained release of functional nanoconjugates aiming to achieve the selective destruction of tumor tissues.

## Data Availability

The data presented in this study are available from Corresponding Author.
